# The Facilitating Role of Chemotherapy in the Palliative Phase of Cancer: Qualitative Interviews with Advanced Cancer Patients

**DOI:** 10.1371/journal.pone.0077959

**Published:** 2013-11-06

**Authors:** Hilde M. Buiting, Wim Terpstra, Floriske Dalhuisen, Nicolette Gunnink-Boonstra, Gabe S. Sonke, Govert den Hartogh

**Affiliations:** 1 Comprehensive Cancer Center The Netherlands, Department of Registry and Research, Utrecht, The Netherlands; 2 University of Amsterdam, Department of Philosophy, Amsterdam, The Netherlands; 3 Onze Lieve Vrouwe Gasthuis, Department of Internal Medicine, Amsterdam, The Netherlands; 4 The Netherlands Cancer Institute, Antoni van Leeuwenhoek Hospital, Department of Medical Oncology, Amsterdam, The Netherlands; Davidoff Center, Israel

## Abstract

**Objective:**

To explore the extent to which patients have a directing role in decisions about chemotherapy in the palliative phase of cancer and (want to) anticipate on the last stage of life.

**Design:**

Qualitative interview study.

**Methods:**

In depth-interviews with 15 patients with advanced colorectal or breast cancer at the medical oncology department in a Dutch teaching hospital; interviews were analysed following the principles of thematic content-analysis.

**Results:**

All patients reported to know that the chemotherapy they received was with palliative intent. Most of them did not express the wish for information about (other) treatment options and put great trust in their physicians’ treatment advice. The more patients were aware of the severity of their disease, the more they seemed to ‘live their life’ in the present and enjoy things besides having cancer. Such living in the present seemed to be facilitated by the use of chemotherapy. Patients often considered the ‘chemotherapy-free period’ more stressful than periods when receiving chemotherapy despite their generally improved physical condition. Chemotherapy (regardless of side-effects) seemed to shift patients’ attention away from the approaching last stage of life. Interestingly, although patients often discussed advance care planning, they were reluctant to bring on end-of-life issues that bothered them at that specific moment. Expressing real interest in people ‘as a person’ was considered an important element of appropriate care.

**Conclusions:**

Fearing their approaching death, patients deliberately focus on living in the present. Active (chemotherapy) treatment facilitates this focus, regardless of the perceived side-effects. However, if anxiety for what lies ahead is the underlying reason for treatment, efforts should be made in assisting patients to find other ways to cope with this fear. Simultaneously, such an approach may reduce the use of burdensome and sometimes costly treatment in the last stage of life.

## Introduction

Major advances in the treatment of cancer characterize the past two decades. An increasing number of treatment options (chemotherapy, endocrine therapy, targeted therapy) in the palliative phase of cancer have become available; some with improved tolerability and therefore available to more general use in cancer patients, also for those in relatively poor condition [1]. Surviving well beyond 2–3 years after diagnosis of advanced disease in chemotherapy responsive tumors (e.g. breast- or colorectal cancer) has become the rule rather than the exception [2], [3]. In the medical community, starting chemotherapy in the palliative phase of cancer for these types of cancer is therefore often considered an ‘obvious thing to do’ and the standard in many oncology guidelines [4].

Survival benefit, however, is not always the (most) appropriate care goal [5], [6]. Chemotherapy is given to delay tumor-related symptoms but may also involve serious side effects and – when death approaches - hinder patients to prepare themselves for death. In addition, second and third line chemotherapy in many malignancies have a limited likelihood of response and only modest improvement in (progression-free) survival, if any [7]. As a result, treatment advances have also confronted physicians with dilemmas concerning the appropriateness of (further) treatment. Chemotherapy in the last weeks/months of life has been described as a potential indicator of inappropriate use of chemotherapy [8]. The trend towards increased use of chemotherapy in the last stage of life [9]–[11] accordingly sparked a debate as to whether physicians should offer such potentially burdensome treatment. Yet, evidence-based information to assess the appropriateness of treatment is scarce and most of the studies originate from the US/Canada. Furthermore, the debate is primarily held at a policy level. On an individual patient level, perceptions towards the appropriateness of treatment may be different. Unfortunately, the patients’ perspective is largely unknown.

In the Netherlands, the Royal Dutch Medical Association (RDMA) launched a checklist for physicians and patients [12] to stimulate an early discussion about the last stage of a patient’s life. It is suggested that - in accordance with existing literature on this topic [13], [14] - such discussions may prevent possible burdensome treatment later on in the disease trajectory. Surprisingly, the (preferred) content of such a discussion as well as patients’ personal ideas about having these discussions is largely unknown. It is important to know whether patients appreciate such discussions, as patients having had sufficient opportunity to discuss their wishes are more likely to receive care consistent with their preferences [15]. It is also important from an ethical perspective: it can be argued that patients living in the face of death need to be informed about their situation to fulfill their (spiritual) needs, encompassing meaning, self-reflection and fulfillment of life-goals [16].

With this study we aimed to explore a) the extent to which patients with potentially chemotherapy responsive tumours (advanced colorectal and breast cancer) have a directing role in decisions about second- and third-line chemotherapy in the palliative phase of cancer and b) already (want to) anticipate on the last stage of life.

## Methods

### Design and Setting

The study described here is the second qualitative part of a larger study that investigates the role of medical professionals in the context of (the limits of) patient autonomy. Qualitative interviews are particularly useful to explore patients’ personal ideas, as they enable respondents to address themes that researchers may not have anticipated [17], [18].

Because of the explorative nature of the study, a qualitative design was considered the most appropriate. In the present study we interviewed patients with advanced colorectal and breast cancer who had received at least one previous line of chemotherapy in the palliative phase of the disease. We chose these cancers because they are chemotherapy-responsive implying that usually more than one line of chemotherapy is considered useful in the palliative phase of the disease.

We define the palliative phase as the time period from the moment that the cancer cannot be cured anymore. With the last stage of the disease, we refer to the last months or weeks of the patients’ life. With treatment, we mean palliative systemic treatment (primarily chemotherapy), unless stated otherwise. For this study, we recruited patients from the medical oncology department of a large teaching hospital in the Netherlands.

### Recruitment and Sampling

We used a purposive qualitative design. With the help of a palliative care nurse who traced patients who were potentially eligible for the study, the oncologists invited patients a) in between chemotherapy lines (the so called ‘chemotherapy-free period’), b) receiving chemotherapy (which could be second/third line chemotherapy), c) not receiving any form of chemotherapy because no reasonably effective chemotherapy was available anymore. In order to reflect the possible diversity of opinions, we further sought for a maximum of variety in treating oncologist, gender (oncologist and patient), patient’s age and stages during the palliative phase. None of the patients refused to participate in the study. In six cases, an informal carer (partner, child) also participated in the interview.

### Interviews

Data for this study were collected from June 2012 to January 2013 through face-to-face semi-structured interviews that lasted on average 40 minutes (minimum 20 minutes/maximum 76 minutes). All except one of the interviews took place at the patients’ home; one patient preferred to be interviewed in the hospital. The treating oncologist explained the study aims and the methods of the study. Next, every potential participant received an information sheet as well as an invitation letter to participate in the study from patient- and physician organizations. If the patient agreed to take part in the study, the patient contact details were given to the primary researcher with substantial interview experience (HMB) [13], [19]–[20]. The primary researcher also introduced and explained the study goals/her role within the study and subsequently arranged a suitable moment to conduct the interview: this was the first time HMB spoke with the patient to be interviewed. HMB conducted all of the interviews and interviews were held until data saturation was reached. We (HMB and FD) discussed the moment data saturation was attained.

We used semi-structured topic lists for the interviews, which were partly based upon study themes common to the debate of possible burdensome treatment at the end of life: **Table 1** provides an overview of these themes referring to present literature. The format was further based on previous qualitative studies [13], [20]. We started with open-ended questions about patients’ experiences with their treating oncologist, their general practitioner and the nurses in the hospital. Next, we asked them specifically to speak about how they themselves experienced and perceived their disease from the moment they had heard that they could not be cured anymore. As far as possible (taking into account the patients’ psychological state-of-mind through careful observation), we tried to address the following topics: what they felt when they had heard that the cancer could not be cured anymore; what is/was meaningful to them in this stage of the disease; their future plans and their experiences of talking about their disease with close relatives and friends and their wishes with respect to the end of life. We subsequently asked them about their treatment experiences. Several issues were explored: the (side) effects of treatment, the process to choose treatment or not, and the patients’ expectations towards treatment. We also studied the patients’ coping strategy and obtained information about the patients’ background such as age, religion and disease history. After every the interview, HMB made some initial field notes. All patients consented to the interview to be audio-taped. The interviews were transcribed verbatim by a professional transcriber; these transcripts were not returned to the patient.

**Table 1:** Study themes common to the debate of burdensome treatment at the end of life.^1^

Theme	Examples
Time trends	Increased use of chemotherapy in the last stage of the patients’ life.
	Increased treatment options in the palliative phase of cancer.
Quality of life	Insufficient knowledge whether possible effects of chemotherapy (palliation, life prolongation) counterbalance the side effects of chemotherapy, especially with respect to second- or third-lines chemotherapy and higher.
	Insufficient knowledge about what is in fact in the patient’s best interest (provision of chemotherapy or not)
Doctor-patient communication	Ambiguous doctor-patient communication in which focusing on chemotherapy may facilitate prognostic misunderstanding.
	Different opinions as to whether cancer can (and should) be defined as a chronic disease.
End-of-life discussions, Death or dying	Not sufficient opportunity for discussion about death and dying.
	Limited information about the (preferred) content of end-of-life discussions.
Early palliative care	Integration of early palliative care early in the course of the palliative disease as an approach that increases the patients’ quality of life and prolongs patients’ survival.
	Limited information about what ‘early’ palliative care precisely involves (as compared to palliative care in the last weeks or months of life).

1 [1], [6], [11], [13], [34], [36]–[41].

### Data Analysis

All data were analysed with qualitative research software (Atlas ti 6.1.12) using a content analysis approach [21]. To increase the validity of the data, HMB and FD both independently read through six interviews (in different time intervals) to identify and code general themes, and subsequently, specific categories within the themes to check for interpreter consensus concerning the assignment of text fragments to major themes:

E.g. for the theme: ‘on and off chemotherapy’ we provided categories such as physical and psychological symptoms and patient’s experiences with respect to the ‘chemotherapy-free period’. Our analysis was ongoing, implying that new themes emerging from the first interviews could be used into subsequent interviews. This led us to add questions about the goals of treatment, the ‘chemotherapy-free period’ as well as patients’ ideas and wishes about the balance between pros and cons with respect to further chemotherapy. We further looked at the text fragments of patients and informal carers separately as well as case studies around one single patient. The data were discussed in several multidisciplinary meetings with people having expertise in health sciences, sociology, ethics, nursing and oncology. In those meetings, we worked towards consensus about the interpretation of key themes. Every time, the primary researcher checked the interpretations with the existing data. WT, GSS and NGB evaluated whether the quotes were used in the right medical context. A professional translator translated the quotes that we eventually chose to illustrate our results. We sent a lay version to the participants who were still alive; the participants could contact the researcher to comment on/ask for clarification.

### Ethical Considerations

The study was approved by the Medical Ethical Committee (VCMO) and written consent was obtained before conducting an interview.

## Results

### Patient Characteristics

The patient sample consisted of patients with an average age of 65 years (see **Table 2**). Half of them were women. Patients were interviewed in different stages of the treatment trajectory but the large majority had had more than one line of treatment and had already discussed the possibility of euthanasia with a physician (general practitioner or oncologist) before the start of the interview. Of the patients who died during follow-up, the time between the last treatment course and the patients’ death varied between 2.5 to 6 months; the time between the patients’ interview and the patients’ death varied between 4 to 9 months. Of the patients who were still alive June 2013∶3 patients stopped chemotherapy because there were no reasonable treatment options anymore; all other patients (5 in total) received some form of (chemo)immunotherapy/targeted therapy after having had one or more lines of chemotherapy.

**Table 2:** Respondent characteristics.

		Patient (n = 15)
Mean age (min, max)		65 (48,85)
Sex	Male	7
	Female	8
Cancer type	Colon	10
	Breast	5
Moment while being interviewed	Indication second line chemotherapy	1
	Second line chemotherapy	4
	Indication third line chemotherapy	1
	Indication third line immunotherapy^1^	1
	Third line chemotherapy	2
	(Indication) third/fourth line chemo-immuno/targeted therapy^1^	2
	Palliative care only	4
Euthanasia discussed when interviewed^2^	Yes	12
	No	3
Importance of religion^3^	Yes (little)	4
	No	11
Presence of someone else during theinterview	Yes, partner	5
	Yes, child/parent	2
	No	8
Number of patients who died afterfollow-up^4^	Yes	7
	No	8

1 In colorectal patients panitumimab (immunotherapy) is a well-accepted approach in the third line [42]; in a selection of breast cancer patients, immunotherapy can be the accepted approach, frequently combined with chemotherapy.

2 The interviewer did not introduce the topic of euthanasia herself: the topic was however frequently mentioned in the context of patients’ (end-of-life) wishes.

3 All patients were asked whether they were having a religious affiliation; during the interview the importance of religion or other life stances sometimes came up, also.

4 Assessed June 2013.

### Qualitative Findings

We identified 4 domains that shed light on, and provided deepened insight into how patients with an advanced form of colorectal or breast cancer perceive and dealt with chemotherapy in the palliative phase of the disease as well as how they anticipated on the last stage of life: the treatment plan in the palliative phase of cancer, disease awareness and living in the present, patients’ physical and psychological condition ‘on and off’ chemotherapy, and patients’ struggles and needs. An overarching theme was patients’ general feeling of being unqualified to make a rational decision to forgo chemotherapy or other form of ‘active’ treatment in the palliative phase of cancer knowing that this could possibly result in having a shorter period of time left.

#### The treatment plan in the palliative phase of cancer

All patients reported to know that the treatment they received was with palliative intent. Patients said that all oncologists clearly indicated that they could not be cured anymore.

*And then X said that the situation was now such that we could forget curative treatment, it now had to be palliative treatment. And then there is a rest period and then progression is looked at […]. So, that was made very clear to me. (Patient 2)*

Oncologists however often remained implicit about their patients’ life expectancy. They tried to reassure the patient, for instance by saying that they would stabilise the disease and maintain a reasonable quality of life or by saying that they could transform this fatal disease into a chronic disease (the definition of a chronic disease was however not further specified). In situations where life expectancy was communicated such openness was sometimes appreciated and sometimes not.

*R: Yes, and then that one [the doctor] told me that with the type of cancer I had - I think it was around seven months to live, and later on the surgeon said to me ‘ X should never have said that, because that’s harsh’.*

*I: And what did you think about it?*

*R: Well I didn’t find it exactly pleasant to hear. Firstly, I was shocked to hear that it had already spread.[…] But now we’re 18 months on, more than 18 months… (Patient 5)*

Patients generally appreciated the way oncologists informed them about their prognosis (e.g. not about life expectancy but about the future course of the disease). They also expressed great trust towards the treating oncologist with respect to the (treatment) plan that subsequently followed. All patients acknowledged to rely on oncologists’ advices with respect to (further) treatment, especially since the treatment decision-making is a delicate process and therefore difficult to comprehend.

*And then people say to you ‘you should look it up on the internet’. Because you can find out anything on the internet of course. But that’s something I don’t do. I get my information from an oncologist and from the GP. And then I wait and see. (Patient 7)*

Most of the patients did not express the wish for information about other palliative (symptom-directed) treatment options while receiving chemotherapy either. Patients seemed to differentiate between the phase in which chemotherapy is provided focusing on treatment, evaluation and hospital visits; and the phase in which chemotherapy or other tumor directed therapy (such as immunotherapy) is not possible anymore and in which the focus is on symptom relief and the approaching death.

*No, but we’re not there yet are we? [Discussion about final phase of life]. It has now been decided that I will get another three courses of the chemotherapy treatment I am getting now, and after the three courses they will look at what sort of effect it has had […]. And if it doesn’t work, then of course you’ll get the discussion … or a discussion… a discussion on ‘what are we going to do now?’ (Patient 2)*

This division could partly be related to unfamiliarity with what palliative care and what end-of-life discussions precisely involved. Patients seemed to associate end-of-life discussions with advanced care planning (e.g. future wishes concerning place of death; end-of-life care and end-of-life decisions). Just as most of the patients did not want to think and speak about their life expectancy, they did not want to speak about the last phase of their life either (the same held for partners or other close carers).

*R2 (partner): […] And we did talk once about the music at the crematorium, […] only, not all at once, but just now and then the subject comes up… for about three or four minutes… but that’s more than enough. (Patient 13)*

#### Disease awareness and living in the present

Yet, patients who expressed awareness that the disease would be fatal sooner or later, acted differently as compared to patients who did not. Although not explicitly pronounced, in interviews with patients for whom awareness of the disease was not evidently present, a general feeling of uncertainty about the (progression of the) disease popped up. Some of the patients tried to keep the cancer as far away as possible and used avoidant coping strategies, for instance by using proverbs. Patients having these difficulties did not want to speak about the approaching last stage of life: they did not even think about discussing it.

*There isn’t really much to talk about. It is what it is and you keep quiet about it. […]. So, no, we haven’t talked about it [death and dying] actually. No. I don’t know why really, it just doesn’t happen. (Patient 1)*

Conversely, patients being aware of their disease seemed to reflect more on the goals of treatment realizing that they could not continue forever balancing the treatment benefits and burdens or balancing the additional value of half a year of life prolongation.

*We have talked about the situation and we said that if I find that the treatments are so gruelling that they are no longer worth going through for the effects they have … and I can’t know this yet … then I want to play a more active role myself and to make the decision for myself when I want to stop treatment. (Patient 4)*

Furthermore, the more the patients were aware of the severity of their disease, the more they seemed to ‘live their life’ and enjoy things besides having cancer.

*[Patient has known since 2004 that the cancer is incurable, the prognosis is now very poor]. Well, the situation is not such that I have to spend all day lying exhausted in bed - that is absolutely not the case […]. What I call quality someone else might not. I spend much of my time here in my study. I also work with groups, drama mainly. (Patient 14)*

All of the patients thus reported to focus on living in the present. Yet, in patients who were not (or did not want to be) aware of the disease, thinking about the approaching last stage of life did not seem to happen. In patients who were aware of the disease, thinking and speaking about the last stage of life did not comply with their wishes: they preferred to ‘live their life’ in the present for which such conversations could be a serious obstacle.

*After hearing ‘oh, but you’re looking great’ and ‘oh, how are you?’ three or four times, by the fifth time of explaining it, it begins to get a bit tiring and I’d really rather not talk about it. [about the disease and about death or dying] (Patient 14)*

Not surprisingly, to imagine forgoing further chemotherapy was even more difficult. Both patients who were aware of the disease and those who were not, indicated that it was hardly possible to specify the hypothetical situation in which they would forgo further treatment, since they realized that stopping would automatically involve progression of the disease and accordingly, shortening of life. Patients only wanted to stop chemotherapy when the side effects of treatment (physically, psychologically) were clearly exceeding the treatment benefits *during* the treatment course.

*I’ll admit it honestly, I’m not strong enough to have already thought ‘no I’m not going to do anything more, I am just going to wait to die’. It’s got something to do with waiting to die. I find it difficult. (Patient 9)*

#### On and off chemotherapy: the patients’ physical and psychological condition

This hypothetical situation seldom seemed to occur in actual practice: regardless of their perception of the severity of the side effects of treatment (although patients remarked that other forms such as targeted therapy were less burdensome to deal with), patients generally accepted them. When directly asked about the side-effects, patients first of all mentioned physical symptoms such as nausea, fatigue, hair loss, neuropathy and lack of energy. The informal carers also reported these side effects.

*Sometimes I gloss over things [during the consultation]…. and I think, ‘it’ll be alright, I’ll get over the side effects’ [of chemotherapy/immunotherapy]. (Patient 13)*

*R2 (partner): Yes, and you are very often tired.*

*R (patient): I am very tired indeed.*

*R2: She stays in bed for probably 20 hours a day, maybe even more.*

*R: Yes, maybe more […]*

*R2: And she can do very little; she can still go out somewhere for about 30 minutes – a short trip to the hospital, or a short ride in the wheelchair, but actually very little. That’s a side effect too. (Patient/partner 11)*

These physical side-effects were frequently associated with psychological side effects of treatment. Patients’ altered appearance because of hair loss could for instance also be very burdensome. In addition, lack of energy could make the many visits to the hospital a very burdensome experience too, especially for older patients or patients living alone.

*No, but I don’t like it. A bald head doesn’t suit me […]. And when it’s all fallen out, people think ‘oh, a cancer patient’. (Patient 14)*

*I was constantly … when I went home then there I was on the phone again…and one day I had to see the oncologist, and then the next day I had to start on the medicines again, […], it drives you to distraction. (Patient 5)*

Interestingly, most of the patients expressed ambivalent feelings concerning the ‘chemotherapy-free’ period. Physically, patients’ condition improved. Psychologically however, patients considered this chemotherapy-free period to be rather stressful because they were aware that the cancer was growing. A large group of patients therefore indicated that CT-scans (during the chemotherapy-free period) were sometimes even more burdensome than the period while receiving chemotherapy.

*The chemotherapy-free period […]. The only thing I think then is ‘well, I feel fine - but because there is no more chemo that thing [tumor] is growing again.’ (Patient 14)*

One patient however reported that for him the ‘chemotherapy-free period’ was the period to relax, to do things, and to fulfill wishes such as the planning of an adventurous holiday.

*Then it was the beginning of November that I had the last chemotherapy treatment, or the end of October maybe. And then just over two months later we went to Thailand. The side effects of the chemo were gone by then so I could do everything. And then I came back and then we decided to fulfill a long-held wish [another wish]; I had always wanted to go to Burma. (Patient 4)*

#### Patients’ struggles and needs

It thus seemed that active (chemotherapy) treatment seemed to shift patients’ attention away from the last stage of life. However, although all patients explicitly remarked that they did not feel like speaking and thinking about the severity of their disease or about death and dying, they indirectly seemed to struggle with their approaching death. This was for instance reflected in how they emphasized their relatively young age. Furthermore, the majority of the patients expressed serious fear for the last stage of life.

*Look, I’m going to die; we all die in the end don’t we? I am also getting older. The thing I am most frightened of is the actual dying phase that is coming. (Patient 5)*

For some patients this was reflected in how they struggled to continue everyday life realizing that they had to reorder/clean things up and that they had to quit particular hobbies. However, both for patients who did (and did not) search for a specific life goal, the uncertainty regarding death and dying was evidently present. This uncertainty seemed to be more intense than the uncertainty of the possible (side) effects of chemotherapy, e.g. translating a decision from everyday life towards a more meta-physical level was rather difficult.

*Well, OK. There could indeed be two reasons for thinking ‘do I like that book [patient is writing a book] so much’ and ‘do I think that this project [work] is so marvelous that I would have chemotherapy so that I might live a little bit longer (you never know). I cannot make up my mind, no. No, I simply cannot make my mind up about it. (Patient 9)*

Interestingly, it seemed that euthanasia was not regarded as a very sensitive topic for these patients, but rather a procedural – semi-judicial - issue. Patients generally consulted the GP to ensure that (s)he was willing to perform euthanasia - if needed.

*No. No contact at all actually [with GP]. I did ask once - I think that it would be about 6 months ago now - if he did euthanasia, if I were the one to ask and if the time had come, and he answered ‘yes’. (Patient 14)*

Patients’ reluctance to speak about end-of-life issues that bothered them at that specific moment may explain why patients reported to appreciate talks with healthcare professionals on topics which were not directly related to ‘cancer’ or ‘death and dying’. This was for instance reflected in how patients sometimes looked for someone expressing interest in medical issues, not directly related to cancer.

*No, the only thing that sometimes… I’ve got a stoma. It gives me a lot of trouble - also mentally - I can’t cope with it actually […]. And it’s not possible to bring up the subject of the stoma with the nurses or with the oncologists actually […]. It is really strange It’s just like they are two different worlds [provision of chemotherapy, taking care of the stoma]. (Patient 7)*

Expressing real interest in people ‘as a person’, e.g. not ‘as a patient suffering from an advanced form of cancer’ seemed to contribute to patients’ well-being. In this context, friends, close relatives, home care, and voluntary services were reported. Equally important, patients further acknowledged that oncologists could have these (informal) talks also, depending on how the discussion was started.

*But the discussion has its own dynamic. It depends on how X begins it - the oncologist – that dynamic, because X has taken the initiative it means that you are next as it were. (Patient 14)*

## Discussion

Our study showed that patients with advanced colorectal and breast cancer know that the chemotherapy they receive has a palliative intent. Patients also seem to use such treatment to cope with fear for their approaching death: regardless of the side effects and patients’ awareness of the disease, chemotherapy appeared to facilitate living in the present. As a result, some of the patients reported that chemotherapy-free periods appeared to be more stressful than periods while receiving chemotherapy. Thinking about forgoing treatment in a later stage of the disease was therefore rather difficult. Patients greatly appreciated physicians’ interest in their life ‘as a person’, which they considered an essential element of appropriate care.

### Strengths and Weaknesses

Study themes common to the debate of possible burdensome treatment at the end of life (see **Table 1**) frequently neglect the patients’ perspective. This study provides valuable information about patients’ perceptions and what stuck in their memory. So, this study should not be seen as a description of actual practice, but as a description of how *patients* experienced the palliative phase of cancer in the context of chemotherapy. We interviewed advanced cancer patients with chemotherapy responsive tumors (breast- and colorectal cancer) in different stages of the treatment course to be able to offer insight into patients’ ideas and experiences in the broadest sense. Our study also has limitations. We only included patients with advanced colorectal- and breast cancer who had undergone one or more previous lines of chemotherapy. Possibly, patients with malignancies with worse prognosis (e.g. non-small cell lung cancer, pancreatic cancer) have different views with respect to death and dying and (continuation) of chemotherapy than other patients. However, we were primarily interested in the ideas and decisions of patients who had already started chemotherapy because stopping is considered to be more difficult than withholding treatment [19], [22].

In addition, because the patients already received treatment, they knew what they were talking about and unrealistic ideas about chemotherapy therefore seem unlikely. We further did not explicitly differentiate between chemotherapy and other forms of palliative systemic treatment although patients sometimes reported that other forms of treatment, such as targeted therapy, were generally less burdensome to deal with. During the interviews, we also realized that informal carers (partner, children) who participated in the interview could be more open and willing to speak as compared to the patients themselves. Yet, we were primarily interested in the patient perspective and we therefore asked the patients how they preferred to be interviewed (accompanied or not). Finally, we only included one hospital. Possibly, our results might be influenced by the department culture. However, we were primarily interested in how *patients* wanted to be involved in the decision-making and wanted to anticipate on the last stage of life; the study focus is thus much more about intrinsic patient values than on doctor-patient interaction. We therefore believe that our findings provide valuable insight into the patients’ perspective relating to chemotherapy in the palliative phase of cancer.

### Discussions about Death and Dying: Future and Present End-of-life Issues

The usefulness of discussions about death and dying in patients with a life-threatening disease has received much attention [23]–[25]. End-of-life discussions in an early stage of the disease have been shown to decrease aggressive care in the last phase of life and improve patients’ quality of life. However, an end-of-life discussion is often operationalized by advance care planning [14], e.g. *future* end-of-life issues. Our study showed that cancer patients relatively easily discussed advance care planning (euthanasia, preferred end-of-life care, place of death) but in fact struggled with *present* end-of-life issues, for instance, on how they should cope with their approaching death at that specific moment.

Perhaps, patients do not know or cannot find the right words to express their concerns. This is partly in line with studies on spirituality that show that patients often struggle to express their spiritual needs and so experience poor quality of life, especially in western countries where religion has largely disappeared [16], [26]–[27]. Perhaps, patients just do not *want* to discuss their concerns because discussing it is considered to be even more burdensome. Our findings at least suggest that discussing future end-of-life issues *only* is insufficient to adequately relieve cancer patients’ distress with respect to death and dying in the palliative phase of cancer. Our findings further suggest that discussions about future end-of-life issues do not automatically facilitate the switch towards symptom palliation, partly because patients who are aware of the disease and actually do talk about future end-of-life issues at the same time prefer to live and enjoy the present, which is facilitated by the use of chemotherapy.

### Chemotherapy: Passive in the Treatment Decision-making, Active in Preventing Death

Patients’ passive stance in the treatment decision-making is to a certain extent remarkable given the tremendous attention that is paid to implement shared decision-making in the clinical encounter [28]–[30]. It can be argued that shared decision-making has to be the norm since it respects patients’ autonomy and gives patients the opportunity to balance the (measurable) treatment benefits and burdens. Simultaneously, it can be argued that patients sometimes prefer a physician-driven decision-making process themselves; especially in this vulnerable stage of life [31]. This second interpretation seems to be more in line with our study findings. However, given the fact that patients deliberately indicated that they did not want to be actively involved in the treatment decision-making patients’ autonomy was well respected. So, viewing patient’s autonomy as a right, our study shows that being passive in the decision-making is not associated with neglecting patient’s autonomy. It is nevertheless unclear whether patients would still have had this passive stance, if oncologists would have structurally discussed other treatment options too and would have elaborated more on other aspects of palliative care where life prolongation is clearly of secondary importance. Although provided by a palliative care service and not by the treating oncologist, Temel *et al* reported that patients who received palliative care (although not clearly specified) integrated with standard oncologic care scored higher on quality of life than patients who received standard oncologic care only [32].

Patients’ passive stance in the treatment decision-making at the same time resulted in an active stance towards their approaching death: chemotherapy gave patients the opportunity to control their life; to subdivide their life in well-ordered parts; to live in the present; and to live a longer life. Previous literature shows that patients frequently want to attain hope for a longer life with the help of chemotherapy [33]. However, we found no literature demonstrating the facilitating role of chemotherapy to enable living in the present. Our study findings however suggest that patients instead of ‘living a longer life’ took ‘living in the present as long as possible’ as their most important starting point. We found no literature demonstrating the facilitating role of chemotherapy to enable living in the present. We also found no literature whether such living in the present reflects certain aspects of hope. Furthermore – in contrast with previous studies [34] - the patients in our study knew very well that they received palliative treatment. Strikingly, this knowledge appeared to be insufficient reason to seriously consider the forgoing of chemotherapy in a later stage of the treatment trajectory. It thus seems that – in the end - patients’ will to live overrides a rational decision to forgo possibly life-prolonging treatment.

### Research and Implications for Clinicians and Policymakers: where to go?

Cost-effective, high-quality end-of-life care currently receives much attention in global health politics, also with respect to palliative systemic treatment. Today, oncologists acknowledge deviating from the standard protocol taking into account the characteristics of the patient and the tumor; the preferences of the patient; and their experiences concerning possible (side) effects of treatment. Our qualitative findings provide important input for further (prospective) qualitative and quantitative studies to improve insight as to whether and when palliative systemic treatment could be considered appropriate or not. It also stresses the importance to carefully consider the underlying reasons of providing chemotherapy: e.g. if this is anxiety for what lies ahead other possibilities to cope with this fear need to be explored and discussed with the patient. Doctor-patient communication, the concept of hope and tumor-specific data about the use of systemic treatment in the palliative phase of cancer deserves special attention:

Patients do not want to think about the very last stage of life. Kiely and colleagues suggested to present patients with typical-, best case and worse-case life expectancy scenarios, allowing them to balance between realism, hope and uncertainty right from the moment they have heard that the cancer cannot be cured anymore [35]. Such information (even if patients remain passive) could prepare patients to prepare for ‘the worst’ but hope for ‘the best’. On top of that and in line with our study findings, such information could diminish patients’ distress with respect to death and dying.

Patients did not explicitly express their hope for a longer life. Yet, patients’ inclination to focus on enjoyable things in the present or on future procedural issues, indirectly seem to reflect (other) aspects of hope. Although hope as a concept has been widely researched in patients with life-limiting illnesses, taking present or future end-of-life issues as a starting point to explore the concept of hope would add to the present body of literature.

Patients accept the side-effects of treatment almost regardless of the perceived severity. Patients however also sometimes indicated that new treatment options such as targeted therapy were less burdensome to deal with. In addition, maintenance treatment with well-tolerated doses of chemotherapy can be offered until progression of the disease or appearance of toxicity. Possibly, distress of the ‘chemotherapy-free period’ may be lowered with maintenance therapy. Such alternative treatment approaches warrant a reflection upon the appropriateness of continuing such treatment till the very end from an ethical/cost-effectiveness perspective.

### What is Already Known

1. Chemotherapy is given to delay tumor-related symptoms but may also involve serious side effects.
2. Starting chemotherapy in chemotherapy-responsive tumors is considered an ‘obvious thing’ to do.
3. Chemotherapy in the last weeks/months of life has been described as a potential indicator of inappropriate use of treatment.

### What this Study Adds

1. Patients may consider the chemotherapy-free period (in-between courses, in-between lines) to be more stressful than the period when receiving chemotherapy.
2. Apart from living a longer life, chemotherapy facilitates the focus to live in the present.
3. Patients do talk about end-of-life issues that refer to the very last stage of life but neglect to talk about end-of-life issues that bother them at that specific moment.
